# Inter-ring rotations of AAA ATPase p97 revealed by electron cryomicroscopy

**DOI:** 10.1098/rsob.130142

**Published:** 2014-03-05

**Authors:** Heidi O. Yeung, Andreas Förster, Cecilia Bebeacua, Hajime Niwa, Caroline Ewens, Ciarán McKeown, Xiaodong Zhang, Paul S. Freemont

**Affiliations:** Centre for Structural Biology, Department of Life Sciences, Imperial College London, London SW7 2AZ, UK

**Keywords:** ATPase, conformational changes, electron microscopy, mechanism, structure

## Abstract

The type II AAA+ protein p97 is involved in numerous cellular activities, including endoplasmic reticulum-associated degradation, transcription activation, membrane fusion and cell-cycle control. These activities are at least in part regulated by the ubiquitin system, in which p97 is thought to target ubiquitylated protein substrates within macromolecular complexes and assist in their extraction or disassembly. Although ATPase activity is essential for p97 function, little is known about how ATP binding or hydrolysis is coupled with p97 conformational changes and substrate remodelling. Here, we have used single-particle electron cryomicroscopy (cryo-EM) to study the effect of nucleotides on p97 conformation. We have identified conformational heterogeneity within the cryo-EM datasets from which we have resolved two major p97 conformations. A comparison of conformations reveals inter-ring rotations upon nucleotide binding and hydrolysis that may be linked to the remodelling of target protein complexes.

## 2. Introduction

ATPase associated with various cellular activities (AAA) proteins are widespread in all types of cells and use the chemical energy released by hydrolysing ATP to perform various biological functions [1]. One important and ubiquitous member of the AAA family, conserved throughout evolution and essential for cell viability [2], is p97 (Cdc48 in yeast). In complex with a large number of cofactors, it participates in numerous cellular activities throughout the cell cycle [3]. Mutations in human p97 (known as VCP) are linked to neurodegenerative disorders, such as amyotrophic lateral sclerosis [4] and inclusion body myopathy associated with Paget disease of bone and frontotemporal dementia (IBMPFD) [5]. In healthy cells, p97/Cdc48 has been implicated, among other processes, in membrane fusion [6], endoplasmic reticulum-associated degradation [7], nucleus reformation after mitosis [8] and DNA damage response [9]. The key to the functional diversity of p97 is the binding of specific cofactors that direct p97 down different functional paths [10] within the ubiquitin proteasome system implicating p97 as a major regulator of global protein turnover.

p97 is a homohexamer comprising an N-terminal domain (N) and two tandem AAA domains (D1 and D2). X-ray crystallographic studies have elucidated the quaternary structure of the complex at near-atomic resolution [11–15] but have not resulted in a full mechanistic understanding of p97. All existing crystal structures are similar, with D1 and D2 stacked ‘head-to-tail’ in a hexameric double ring and N domains coplanar with D1 irrespective of nucleotide state. One crystal structure of a disease mutant form of p97 shows the N domain slightly above the D1 ring, hinting at the possibility of larger conformational transitions [16]. Solution studies by cryo-EM [17–19] and small-angle X-ray scattering [20] confirm a more dynamic picture of p97. Although the ATPase activity of p97 is primarily contributed by D2, recent studies show that communication of conformational change between the rings and mobility of N domains is important for ATPase activity [21–23]. Consolidation and interpretation of the conformational states of p97 have however remained ambiguous.

Here, we have used a variety of EM techniques to visualize the conformations of p97 in different nucleotide states. By separating protein conformations within the same single-particle dataset, we avoid the conformational variability of p97 that has confounded earlier analyses and are able to identify two primary conformations that exist simultaneously in solution. We show that one of these is highly dynamic throughout the nucleotide hydrolysis cycle: the position of the N domains above or in plane with the D1 ring is linked to different conformations of the D1 and D2 rings. We also measure nucleotide binding-dependent rotations of the D1 ring relative to D2. Our new reconstructions allow us to propose a mechanism that links N-domain position to conformational transitions within D1 and D2 that may be linked to the remodelling of target protein complexes.

## 3. Results

### 3.1. p97 exhibits conformational heterogeneity under electron cryomicroscopy conditions

Cryo-EM and single-particle analysis were used to study the conformation of p97 in different nucleotide states. To eliminate heterogeneity owing to slow ATPγS hydrolysis, a hydrolysis-deficient D2 mutant (p97^E578Q^ [24]) was used throughout. Three datasets were collected, with no added nucleotide, 100 μM ATPγS and 100 μM ADP, respectively.

**Figure 1.**
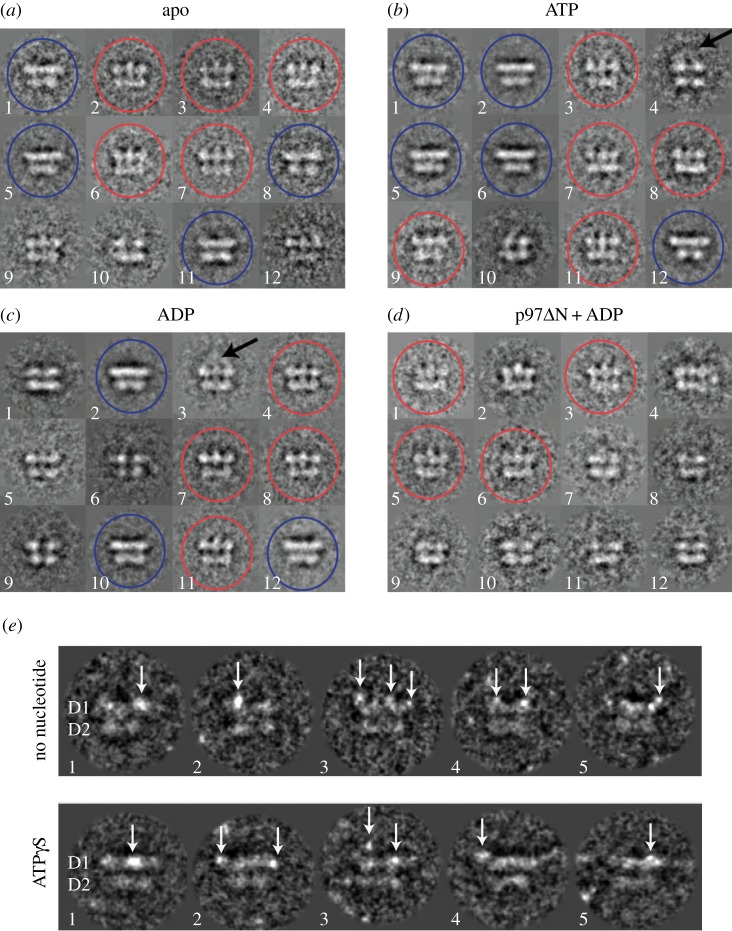
p97 shows conformational heterogeneity. Class averages of around 20 reprojections representing side views of p97 obtained (*a*) in the absence of nucleotide, (*b*) after incubation with ATP and (*c*) after incubation with ADP, as well as (*d*) side views of p97ΔN obtained after incubation with ADP were built using MSA classification. Two different side views were observed in all datasets except p97ΔN. Side view 1 (red circles) shows three distinct densities for the top layer. Fuzzy density can occasionally be seen above the top layer, suggesting flexible N domains (arrows). Side view 2 (blue circles, not observed in p97ΔN) features flat, continuous densities for both layers. (*e*) Nanogold particles binding to the His-tag at the N-terminus of full-length p97^E578Q^ appear as bright spots (arrows) in projection images. Projections representing side views of p97 were extracted for this figure. Two layers can be seen in each projection. The top layer corresponds to the D1 ring, whereas the bottom layer corresponds to the D2 ring. In the absence of added nucleotide, nanogold particles were seen mainly above the D1 ring (top row), while in the presence of 100 μM ATPγS, nanogold particles can occasionally be seen coplanar with the D1 ring (bottom row, views 1 and 2).

Upon examining class averages of projections representing side views of p97, we identified two major types within all three datasets (figure 1*a–c*). The first type (side view 1) features three distinct densities at the top layer and a continuous density at the bottom layer. The second type (side view 2) features two layers of flat, continuous density, with a wider top layer. In class averages of side view projections of p97 without the N domain (p97ΔN), only side view 1 was observed (figure 1*d*). Together, these data suggest that differences in side view projections are owing to N-domain conformations. To confirm this, we used Ni-NTA-Nanogold to label the N-terminal His-tag of full-length p97 (figure 1*e*). Regardless of nucleotide state, we observed nanogold particles above (figure 1*e*; top row, views 2, 3, 4, and bottom row, view 3) or coplanar (figure 1*e*; bottom row, views 1, 2, 4) with the D1 ring. Occasionally, we also observed differences in the position of the gold particles within one p97 molecule (figure 1*e*; bottom row, view 2). These results indicate that p97 can adopt multiple conformations and that the relative position of the N domains is a key feature of this heterogeneity.

To investigate whether conformational changes are induced by nucleotide binding to D2, we determined the stoichiometry of prebound nucleotide in purified p97. Our previous studies showed that approximately 90% of the D1 domains of wild-type p97 contain ADP, whereas D2 is free of bound nucleotide [25]. Using the previously described heat denaturation protocol, we found that 1.1 ± 0.1 nucleotides prebound per p97^E578Q^ monomer, similar to data for wild-type p97 [26]. Given the differential binding affinities between D1 and D2 for ADP and ATPγS [25], this supports a model where the D1 ring is fully occupied with ADP and additional nucleotide, as added in the EM experiments, is bound to D2.

### 3.2. Three-dimensional reconstructions of p97 in apo, ATPγS and ADP states

To separate p97 particles within the cryo-EM data, two initial models were built from the two different side views and used as references for competitive alignment within each dataset (see Material and methods). Particles aligning to either reference were sorted into different subsets. Data processing and refinement was subsequently carried out using standard angular reconstitution procedures.

**Figure 2.**
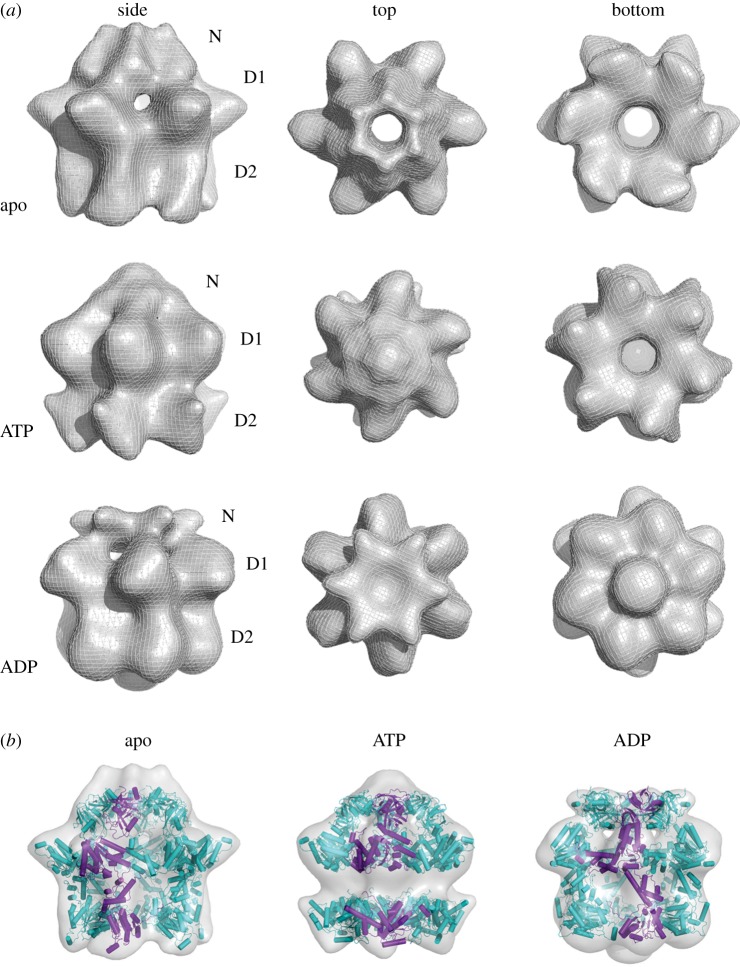
Three-dimensional reconstructions of p97^E578Q^ in different nucleotide states—conformation 1. (*a*) Conformation 1 has a three-layered architecture with the top layer assigned to the N domains, and the middle and bottom layers to the D1 and D2 domains, respectively. The top row is the apo state, the middle row the ATPγS state and the bottom row the ADP state. The first column shows side views, the second column top views and the third column bottom views. In all three reconstructions, the D1 ring is expanded, while conformational changes can be seen in the D2 ring depending on the nucleotide state. The N domains appear above the D1 ring as a tall dome (apo state), an intermediate conformation (ATPγS) or a flat cap (ADP state). (*b*) Fitting of p97 as individual N, D1 and D2 domains shows overall compatibility of the cryo-EM reconstructions with the atomic model of p97.

**Figure 3.**
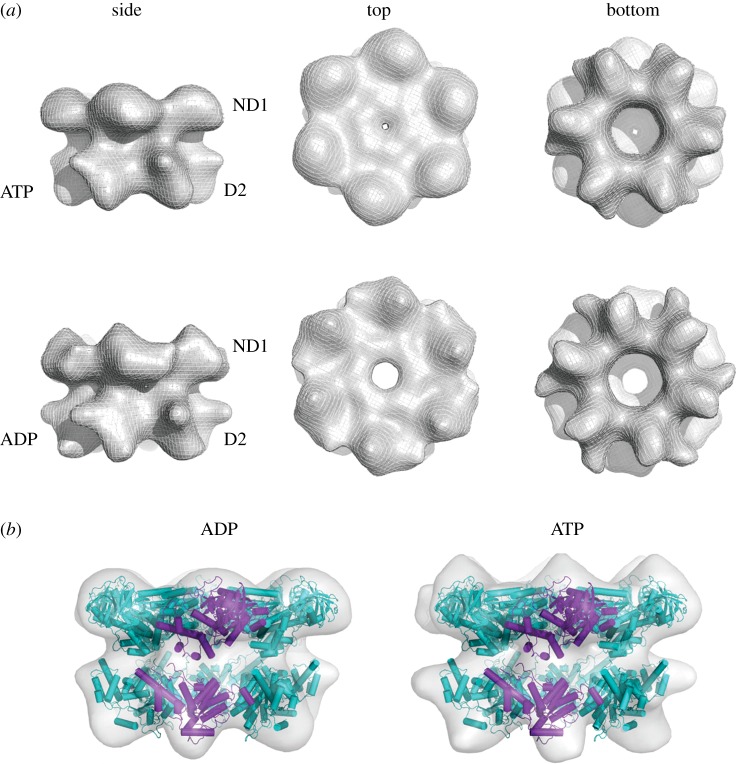
Three-dimensional reconstructions of p97^E578Q^ in different nucleotide states—conformation 2. (*a*) Conformation 2 has a two-layered architecture with the top layer assigned to the N and D1 domains, and the bottom layer to the D2 domains. The top row is the ATPγS state and the bottom row the ADP state. The first column shows side views, the second column top views and the third column bottom views. In both nucleotide states, conformation 2 is similar to published crystal structures of p97. There are only small differences between the ADP and ATPγS reconstructions. (*b*) To fit p97 into the cryo-EM reconstructions of conformation 2, only slight changes have to be made to the crystal structure of p97.

Two distinct p97 conformations were identified in all three nucleotide states (figure 1). In the presence of nucleotide, both conformations have similar prevalence. In the absence of nucleotide, conformation 1 dominates. In conformation 1, the N domains are located above the D1 ring (figure 2*a*), whereas they are coplanar with the D1 ring in conformation 2 (figure 3*a*). The final reconstructions of conformation 1, at resolutions between 17 and 20 Å, show similar three-layered hexameric structures (figure 2*a*). The top layer occupies the least density and was assigned to N domains. Strongly connected density that rises in a tall dome can be seen for N domains in the apo and ADP states, whereas a flat cap is observed above the entrance to the D1 pore in the ATPγS state (figure 2*a*). The D1 and D2 rings form the middle and bottom layers of the reconstructions, respectively. All three structures show an expanded D1 ring with pronounced hexameric protrusions and a wide central pore. The D2 ring is narrower and shows large nucleotide-dependent changes (figure 2*a*), most notably in the orientation of the six D2 domains relative to the central axis of p97.

Conformation 2 is primarily observed in the presence of nucleotide. Forty per cent of particles imaged in the presence of ATPγS or ADP but only about 5% of the particles in the apo dataset aligned to this conformation. Thus, only the reconstructions in conformation 2 will be described for the ADP and ATPγS states. Refinement resulted in final structures of approximately 15 Å resolution comprising a two-layered hexameric barrel with a wide top layer and narrower but more open bottom layer (figure 3*a*). There is little conformational difference between the ATPγS and ADP structures. The N domains are coplanar with the D1 domains forming a relatively flat ND1 ring similar to what is observed in p97 crystal structures. The D2 ring is more open than that of conformation 1.

### 3.3. Docking of the p97 crystal structure into electron cryomicroscopy conformations

EM reconstructions obtained from transmission electron microscopic images have ambiguous handedness, a limitation inherent in the method. The correct handedness of all reconstructions in this work was determined by comparison with the known X-ray structure of p97. Specifically, helix 3 in the ATPase domains protrudes from the core of the domain, giving the domain the overall shape of a comma that curves clockwise (CW) when viewed from the top of p97. Clockwise curvature of the protrusions of the ATPase domains disambiguates the hand of all reconstructions (electronic supplementary material, figure S1). A model derived from the p97 crystal structure fits better into reconstructions of the correct hand than into those of the opposite hand.

A hybrid p97 protomer (see Material and methods) was manually placed inside the density of each of the five EM reconstructions. This initial position was then reciprocal-space refined with sixfold symmetry constraints. Fitting p97 into conformation 1 requires a large rearrangement of the N domains relative to the D1 ring. These rearrangements are modelled, in the different nucleotide states, by rotations that flip the N domain into an apical position and are constrained by the N–D1 linker (figure 2*b*). In addition, the D1 ring is expanded in all nucleotide states, with individual subunits rotated outward and up. The D2 ring also expands somewhat relative to the crystal structure, but this change is minor compared with that in the D1 ring.

Fitting was straightforward for conformation 2, whose flat top layer shows a significant resemblance to the ND1 ring in the p97 crystal structures (figure 3*b*). The N domains required a slight upward rotation, but not nearly to the extent that is seen in the structure of an IMBPFD mutant of p97 [16]. To obtain a good fit for the D2 ring, individual domains had to be rotated considerably downward and slightly CW (as seen from the top). This results in an opening up of the D2 ring. In addition, instead of inserting tightly into depressions between neighbouring D1 domains as in the crystal structures, the D2 domains in conformation 2 are removed from the ND1 ring, packing loosely against the basal protrusions of D1 domains. The larger separation between the ND1 and D2 rings can be accommodated by the long flexible linker between D1 and D2 (residues 440–480).

### 3.4. Comparison of p97 reconstructions

**Figure 4.**
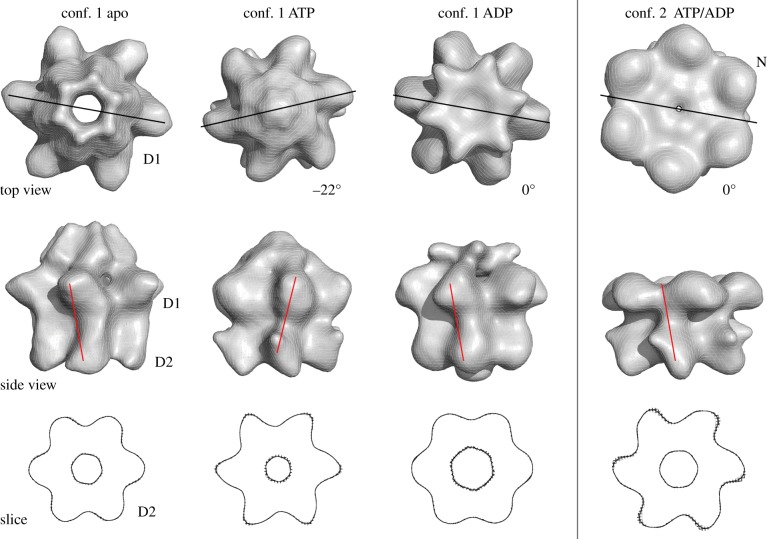
Rotation of the D1 ring with respect to D2. The top views in the top row show how the D1 ring rotates with respect to the D2 ring in the ATPγS state. Lines indicate the relative position of a D1 domain relative to a D2 domain, and numbers reflect the rotation of the D2 ring with respect to its position in the apo state. In conformation 1 in the presence of ATPγS, the rotation of the D1 ring relative to D2 is 22° CCW. There is no rotational difference between the ADP state and the apo state. In conformation 2, in the presence of nucleotide, there is no rotation of the D1 ring relative to D2 when compared with the apo state of conformation 1. The middle row shows side views of p97. The lines indicate the relative position of a D1 domain with respect to D2. The alignments were prepared by superposition of the D2 rings among the different conformations and nucleotide states, as demonstrated by slices through the D2 rings of the reconstructions, shown in the bottom panel. In all three rows, the four columns show, from left to right, conformation 1 in the apo state, conformation 1 in the ADP state, conformation 1 in the ATPγS state and conformation 2 (nucleotide bound), respectively.

The presence of nucleotide leads to a change in the conformation of the N domains. Whereas they assemble to extend the central pore in the apo structure, they occlude it in the ATPγS and ADP states of conformation 1 and are locked coplanar to the D1 ring in conformation 2 (figure 4). The D1 rings of the three reconstructions in conformation 1 share pronounced radial protrusions and exhibit high degrees of overall similarity, indicating a stable conformation of D1 irrespective of nucleotide state. By contrast, there are significant changes in their D2 rings. In the ADP state, the ring is compact but with a wide central pore that is occluded by basal density, possibly indicating the position of the C termini. In the ATPγS state, the D2 domains appear sharply tilted with conspicuous radial protrusions. Conformation 2, which is very similar in both nucleotide states, seems to share the orientation of the D2 domains with the ATPγS state of conformation 1.

A comparison of the relative arrangements of the D1 and D2 rings in the different nucleotide states and conformations reveals striking rotations (figure 4, top and middle rows). For comparison, we align the reconstructions by their D2 rings (figure 4, bottom row). In conformation 1, the ATPγS state (a proxy for ATP binding) differs from the apo state by a 22° counterclockwise (CCW) rotation of the D1 ring, as seen from the top. There is no difference in relative ring rotation between the ADP-bound and apo state. The relative rotation between the rings in the ATPγS state in conformation 2 (measured under exclusion of the N domains; see the electronic supplementary material, figure S2) is unchanged from that of the apo form in conformation 1.

## 4. Discussion

### 4.1. The N domains of p97 are highly dynamic

Previous cryo-EM reconstructions of p97 in different nucleotide states show poor correlation, making it difficult to understand the conformational changes caused by nucleotide binding and hydrolysis. Here, separation of p97 particles based on different side views has resulted in the extraction of two major conformations. Positioned either coplanar with or in various positions above the D1 ring, the N domains are a major source of p97 conformational heterogeneity irrespective of nucleotide state, and two major p97 conformations can be described based on the position of the N domains. Conformation 1 shows p97 hexamers with poorly defined density above the hexamer ring, which we attribute to mobile N domains. Conformation 2 shows N domains coplanar with the D1 ring and in apparent rigidity, similar to the p97 crystal structures, whereas the D2 ring appears more open.

Our improved cryo-EM reconstructions of p97 thus indicate a previously unappreciated degree of conformational flexibility that is not restricted to the N domains but is apparent in D1 and D2. There is clear cooperativity among the three sets of domains. In conformation 1, the rearrangement of individual D1 protomers with respect to the crystal structures results in an open D1 pore and a raised position of the N domains. In addition, the D2 domains show various degrees of tilt. In conformation 2, the N domains are coplanar, the D1 ring is tightly packed and the D1 pore closed, but the D2 pore is more open and the D2 domains are less tightly packed. Together these observations suggest that N domains are conformationally coupled to the ATPase domains.

In conformation 1, application of C6 symmetry during the reconstruction process resulted in an averaging of densities attributable to flexible N domains. Although we can model N domains into this density, these static models are not representative of the situation in solution. Similarly, asymmetry within the D2 ring caused by incomplete nucleotide binding to the six protomers, which previous biochemical studies have demonstrated [25], is not captured here. Our reconstructions thus represent a simplified picture of p97 flexibility. Nevertheless, they emphasize the flexible nature of N domains, which is particularly relevant to p97 function given that the position and mobility of the N domains are related to ATPase activity [21,27], and that p97 interacts with cofactors and consequently targets ubiquitylated substrates through N domains. For example, the cofactor Ufd1–Npl4 was shown by cryo-EM to assume various positions relative to the D1 ring, consistent with N domain flexibility [28]. N domain repositioning as a function of nucleotide binding or hydrolysis may thus provide the necessary force for p97 to disassemble or unfold target proteins.

### 4.2. Nucleotide-dependent conformational changes in p97

Our reconstructions of p97 have also captured different conformations for the D1 and D2 rings. As conformation 2 is almost absent from apo state, and similar in appearance for the ATPγS and ADP states, it is appealing to consider it a generic nucleotide-bound state, which may be in equilibrium with conformation 1 nucleotide-bound states. The differences between these states would be the repositioning of the N domains from flexible positions above D1 to D1-coplanar positions. Conformation 2 is nearly identical in the ATP- and ADP-bound states, and no nucleotide-dependent inter-ring rotations are observed when N domains are locked coplanar with D1. By contrast, for conformation 1, where the N-domains are flexible, we can observe inter-ring rotations upon ATP binding that are consistent with what was observed in high-speed atomic force microscopy (AFM) experiments that showed in real-time rotation of the D1 ring with respect to D2 upon the addition of ATP [29].

In the AFM work, rotation was measured relative to the C termini of p97, which were immobilized on a mica surface. We took sections approximately halfway through the D2 ring as the point of reference. As p97 is not a straight molecule but shows distinct handedness, it is not surprising that the extent of rotation observed in AFM (23° CW) and in our work (22° CCW) differs. To ensure that our conclusions are not an artefact of a poor alignment, we also aligned our maps on their D1 domains. We still measure a rotation in the ATP-bound state of a bit more than 20° (electronic supplementary material, figure S3 and movies S1–S3), and obtain nearly identical ring rotations for the apo and ADP states. (The direction of rotation is inverted because the motion is now relative to D1 and not to D2.) Morphs between pairs of reconstructions show that the transition to and from the ATP state goes along with a D2 domain transformation that repositions a conspicuous protrusion that we annotate as helix 3 (electronic supplementary material, movies S4–S6). In summary, both our study and the earlier AFM work are in agreement that binding of ATP but not ADP induces the conformational changes in the D2 domain. Our data are furthermore consistent with the hypothesis that the location of the N-domain is coupled to inter-ring rotations upon ATP binding and hydrolysis.

Because of the periodicity in a hexamer of 60°, a 22° CCW rotation and a 38° CW rotation leads to equivalent relative orientations of the D1 and D2 rings. Neither direction of rotation is implausible. On the one hand, the 22° CCW rotation appears to preserve inter-domain connectivity (figure 4, middle row). On the other hand, a 38° CW rotation would bring the linker between D1 and D2 near an adjacent D1 domain (figure 4, middle row), supporting a previously proposed mechanism to explain the communication of conformational change from D2 to the D1 domain through the linker of the neighbouring but not same protomer [22]. To disambiguate, higher-resolution cryo-EM data or labelling of an individual subunit in high-speed AFM would be required.

In N-ethylmaleimide-sensitive factor (NSF), an AAA ATPase related to p97 that disassembles the soluble NSF attachment protein receptor (SNARE) complex in an ATP-dependent manner, D1 rotates CCW relative to the D2 ring after γ-phosphate release [30]. The approximately 22° rotation induced by ATP hydrolysis was used to suggest a mechanism for converting the chemical energy of ATP into mechanical work. While the specifics (direction of rotation, catalytically active ATPase domain, ATP binding versus ATP hydrolysis) may differ, the mechanisms underlying the disassembly of protein complexes by NSF and p97—conversion of repeated rotations to mechanical work—are likely to be fundamentally similar.

It is tempting to speculate that conformation 2 represents the entrance point into ATPase cycle. In earlier experiments, when p97 was held in what amounted to conformation 2 (N domains chemically cross-linked to be coplanar to the D1 ring), ATP hydrolysis was suppressed [21], which strongly argues that hydrolysis is connected to a switch from conformation 2. We suggest that transition between conformations 1 and 2 takes place *in vivo*, either as a means of sampling conformational space or to probe the environment for interacting partners that are required for the biological function of p97. In the absence of nucleotide, conformation 1 predominates. Presence of nucleotide stabilizes conformation 2. Upon binding of ATP and repositioning of the N domain–adaptor subcomplex, ATP hydrolysis starts. The N domains are released and the D1 ring rotates back against D2 into the position observed in the apo state.

## 5. Conclusion

Our structures provide global information on relative motions within the p97 hexamer that are consistent with various existing cryo-EM and crystal structures, and with recent single-molecule observations by high-speed AFM. The data indicate coordinated flexibility in the N as well as the ATPase domains, and suggest a possible way of converting the chemical energy stored in ATP into mechanical energy required for the remodelling of substrates. Given the conformational heterogeneity within p97, more detailed structural characterization by single-particle cryo-EM (e.g. at the level of individual protomers) will require further breakthroughs in methodology. For now, it seems clear that the biological function of p97 requires positioning of the N domain coplanar to D1 and relative rotations of the ATPase rings.

## 6. Material and methods

### 6.1. Protein expression and purification

Full-length p97 (N-terminally His_6_-tagged E578Q mutant) was expressed as described for wild-type p97 [28], except that the affinity buffers contained 5% glycerol, protein was eluted in 100% buffer B and the gel filtration buffer contained 250 mM NaCl. Fractions containing p97^E578Q^ were pooled and concentrated to 5 μM.

### 6.2. Nanogold labelling

Samples containing purified p97 were incubated on ice for 30 min with Ni-NTA nanogold (Nanoprobes Inc.) at a ratio of one p97 hexamer to one labelling particle. In samples requiring addition of ADP or ATPγS, nanogold labelling was carried out immediately after incubation with nucleotide.

### 6.3. EM data collection

Protein was loaded onto grids, either directly or after incubation with 100 μM of ATPγS or ADP for 30 min on ice, and attached to a Vitrobot (FEI) set to 4°C with a blotting time of 2 s. Data were collected on a Phillips CM200 FEG electron microscope using a 4 k × 4 k CCD camera (TVIPS) at 50 k magnification (1.76 Å pixel^−1^).

### 6.4. Image processing

Image processing was primarily carried out in IMAGIC [31]. Images were coarsened and CTF-corrected and particles were automatically picked, band-pass filtered, normalized and centred. The APO, ATP and ADP datasets contained approximately 27 500, 46 600 and 30 300 particles, respectively. Class averages were generated using multivariate statistical analysis. As sixfold symmetry was observed in the eigenimages, C6 symmetry was applied throughout. An initial model was built with two class averages corresponding to top and side views and then refined in every dataset using iterative rounds of angular reconstitution [32], three-dimensional reconstruction and brute-force alignment (written by Timothy Grant) followed by rounds of refinement by projection matching [33]. The resolution was estimated by Fourier shell correlation and the 1/2-bit criterion [34].

### 6.5. Fitting of X-ray structures

A hybrid protomer built from p97-ND1 [14] and the D2 domain of full-length p97 [15] was roughly placed inside the densities corresponding to the five p97 reconstructions with PyMOL (http://www.pymol.org). For all five reconstructions, the domains comprising the hybrid protomer (N, D1, D2) were manually and separately adjusted to obtain a good initial fit constrained by the EM densities and the relative orientation of the domains. Adjustments were as small as possible and within the reach of the N–D1 and D1–D2 linkers. These initial fits were then refined in VEDA (http://mem.ibs.fr/VEDA/) using reciprocal-space refinement with sixfold symmetry imposed.

### 6.6. Creation of movies

Movies were created in Chimera [35]. First, maps were manually aligned on their D1 domains. This fit was improved using the ‘Fit in Map’ tool. The morphs between the fitted maps were created with the ‘Morph Map’ tool and saved as mp4 files.

## Supplementary Material

Supplementary Electronic Material

## Supplementary Material

Sup_Figures

## Data accessibility

The cryo-EM maps have been deposited in the EM data bank (http://www.ebi.ac.uk/msd/) under accession codes EMD-2589 (p97-apo-conf1), EMD-2590 (p97-ADP-conf1), EMD-2591 (p97-ATPγS-conf1), EMD-2592 (p97-ADP-conf2) and EMD-2593 (p97-ATPγS-conf2).
