# Supplementary material for: Inter-ring rotations of AAA ATPase p97 revealed by electron cryomicroscopy
Source: Open Biol. 2014 Mar 5;4(3):130142. doi: 10.1098/rsob.130142 (PMC3971404; doi:10.1098/rsob.130142)
Supplement: Supplementary Electronic Material [file rsob130142supp1.docx]

Supplementary Electronic Material

**Figure S1: Determination of the handedness of the p97 reconstructions.** (a) Determination of the handedness of conformation 2 (right) is facilitated by the high degree of similarity to the model used for fitting (left). Especially helpful are the curvature of the D2 domains (top, red arrow) and a long helix in D2 (bottom, red bar). (b) The D2 domains of conformation 1 in the ATP state show the same curvature (top, red arrow) and the same protrusions (bottom, red bar) as is observed in conformation 2. (c) The curved appearance of the D1 domains in the apo state in conformation 1 enables identification of the correct handedness (red arrow). This conformation is consistent with the appearance of the D1 domains in the molecular model. (d) In conformation 1, the D2 domains in the ADP state look similar to those in the apo state and the D1 domains show similar, though less pronounced, curvature.

**Figure S2: Rotational state of the D1 ring in conformation 2.** The N domains in a coplanar conformation obfuscate the position of the D1 domains. To identify the most peripheral part of D1, which is used for determining the rotational state, one has to remove the N domains. (a) Surface representation of the hybrid model of p97 (used for fitting) inside the envelope of conformation 2 showing the N domains in salmon. This top view is oriented like the reconstruction in panel 4 of Fig. 4. (b) Surface representation of truncated p97 whose N domains were removed *in silico*. The view is the same as in (a). The protrusions corresponding to the D1 domains are not congruent with the N domains.

**Figure S3: Rotation of D2 rings relative to D1.** For this figure, the p97 maps were aligned on their D1 rings. The ATP state (middle panel) shows a relative rotation with respect of the apo state (left panel) of around 25°. This corresponds to a -25° rotation relative to the D2 rings, similar to what was measured when the maps were superposed on their D2 rings (Fig. 4). The ADP state (right panel) looks very similar to the apo state.

**Movie S4: Ring rotation between apo and ATP states in conformation 1.** After superposition of the maps on their D1 rings, a horizontal slice through the D2 rings is shown in this movie. The view is down from the top of p97.

**Movie S5: Ring rotation between ATP and ADP states in conformation 1.** Orientation as in Movie S1.

**Movie S6: Ring rotation between ADP and apo states in conformation 1.** Orientation as in Movie S1.

**Movie S7: Transition between apo and ATP states in conformation 1.** Map superpositions were created as for Movie S1, but p97 is shown in a side view.

**Movie S8: Transition between ATP and ADP states in conformation 1.** Orientation is as in Movie S4.

**Movie S9: Transition between ADP and apo states in conformation 1.** Orientation is as in Movie S4.
