# Supplementary figures and images for: Inter-ring rotations of AAA ATPase p97 revealed by electron cryomicroscopy
Source: Open Biol. 2014 Mar 5;4(3):130142. doi: 10.1098/rsob.130142 (PMC3971404; doi:10.1098/rsob.130142)

**Figure S1**

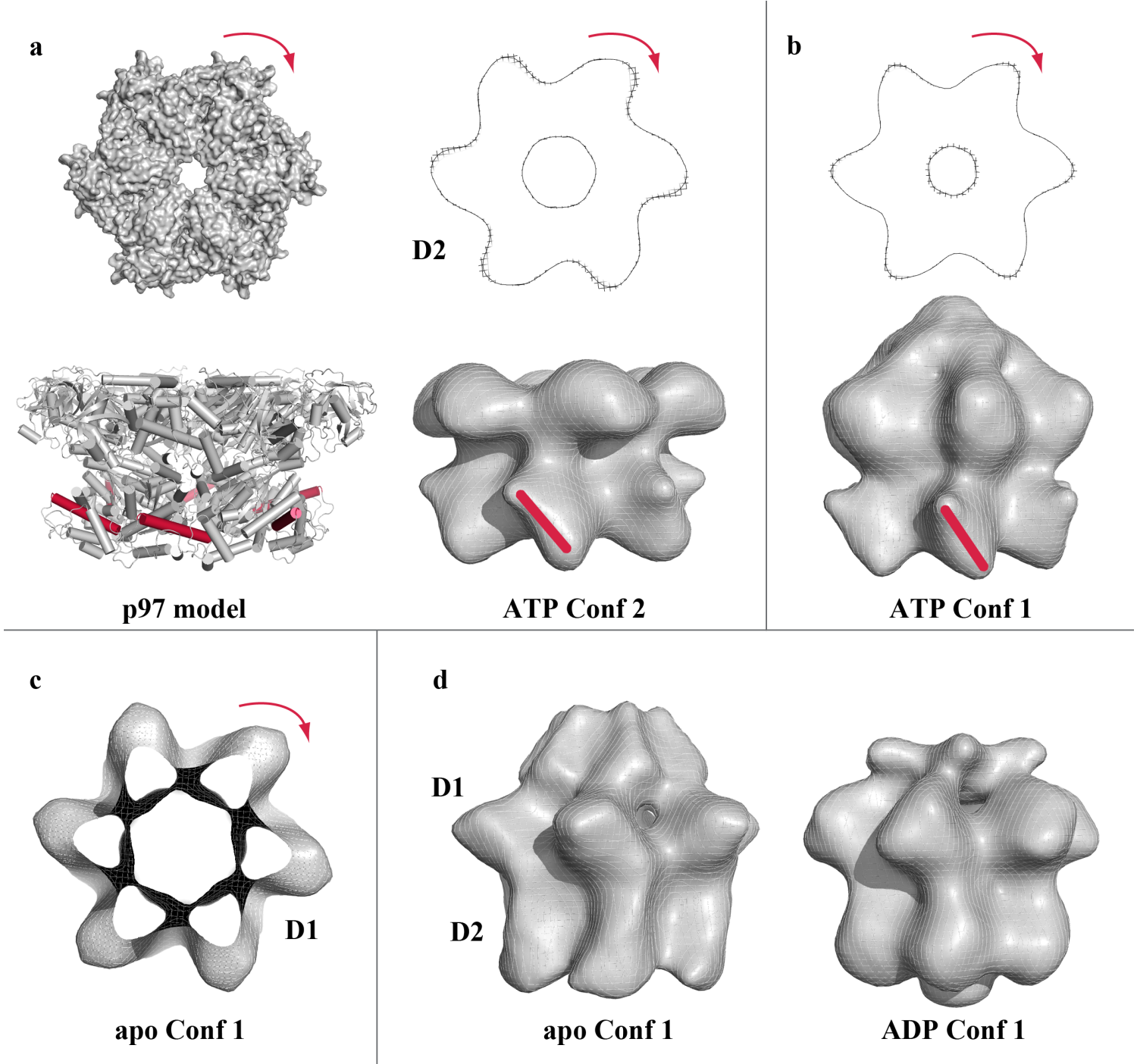

**Figure S2**

**a**

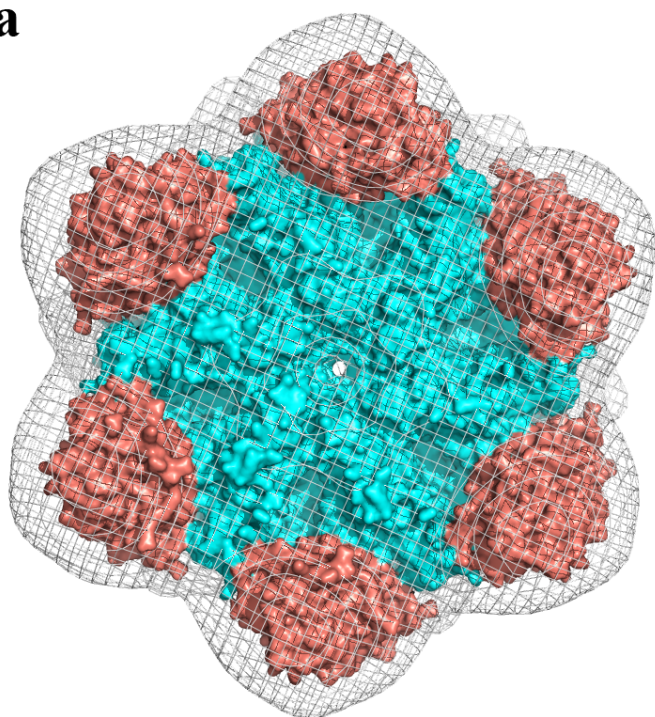

**b**

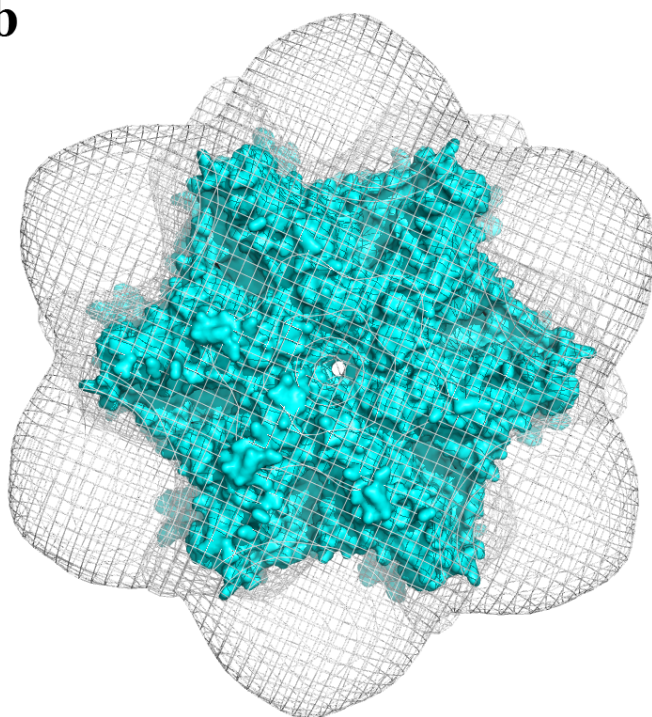

**Figure S3**

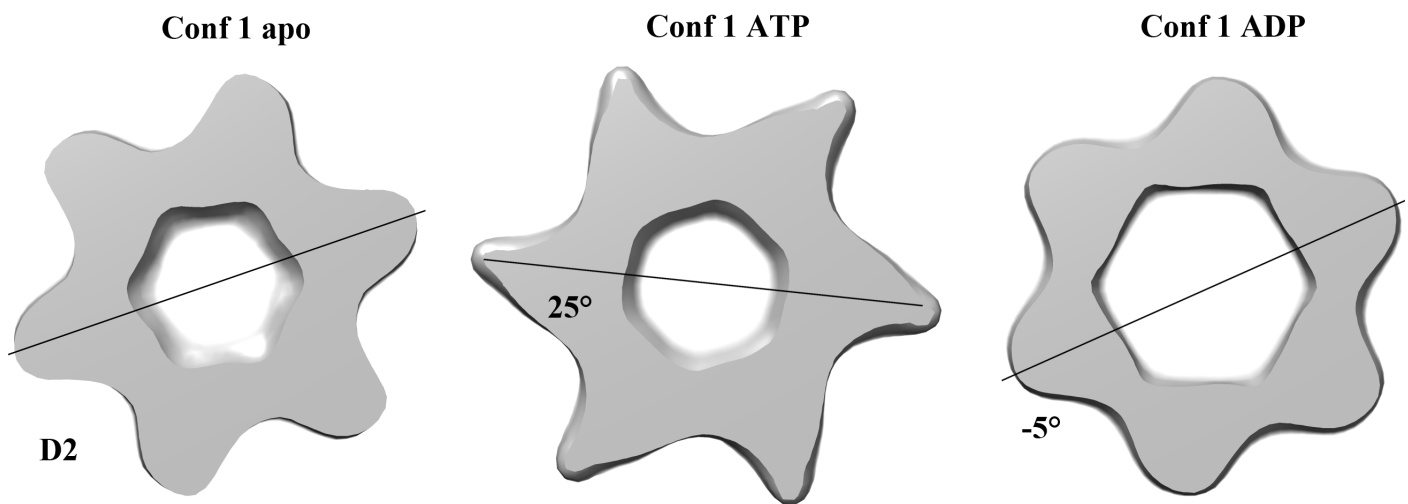

Supplement: Sup_Figures [file rsob130142supp2.pdf]
